# Evaluation of three high abundance protein depletion kits for umbilical cord serum proteomics

**DOI:** 10.1186/1477-5956-9-24

**Published:** 2011-05-09

**Authors:** Bin Liu, Fang-hua Qiu, Courtney Voss, Yun Xu, Ming-zhe Zhao, Yan-xin Wu, Jing Nie, Zi-lian Wang

**Affiliations:** 1 Department of Obstetrics and Gynecology, The First Affiliated Hospital of Sun Yat-sen University, Guangzhou, 510080, PR China; 2 Laboratory of Proteomics, The First Affiliated Hospital of Sun Yat-sen University, Guangzhou, 510080, PR China; 3 Department of Biochemistry, University of Western Ontario, London, Ontario, N6A 5C1, Canada; 4 Department of Endocrinology, The Sixth Affiliated Hospital of Sun Yat-sen University, Guangzhou, 510000, PR China

## Abstract

**Background:**

High abundance protein depletion is a major challenge in the study of serum/plasma proteomics. Prior to this study, most commercially available kits for depletion of highly abundant proteins had only been tested and evaluated in adult serum/plasma, while the depletion efficiency on umbilical cord serum/plasma had not been clarified. Structural differences between some adult and fetal proteins (such as albumin) make it likely that depletion approaches for adult and umbilical cord serum/plasma will be variable. Therefore, the primary purposes of the present study are to investigate the efficiencies of several commonly-used commercial kits during high abundance protein depletion from umbilical cord serum and to determine which kit yields the most effective and reproducible results for further proteomics research on umbilical cord serum.

**Results:**

The immunoaffinity based kits (PROTIA-Sigma and 5185-Agilent) displayed higher depletion efficiency than the immobilized dye based kit (PROTBA-Sigma) in umbilical cord serum samples. Both the PROTIA-Sigma and 5185-Agilent kit maintained high depletion efficiency when used three consecutive times. Depletion by the PROTIA-Sigma Kit improved 2DE gel quality by reducing smeared bands produced by the presence of high abundance proteins and increasing the intensity of other protein spots. During image analysis using the identical detection parameters, 411 ± 18 spots were detected in crude serum gels, while 757 ± 43 spots were detected in depleted serum gels. Eight spots unique to depleted serum gels were identified by MALDI- TOF/TOF MS, seven of which were low abundance proteins.

**Conclusions:**

The immunoaffinity based kits exceeded the immobilized dye based kit in high abundance protein depletion of umbilical cord serum samples and dramatically improved 2DE gel quality for detection of trace biomarkers.

## Background

The fetal period is an important and special time in human life, in which the fetus grows quickly and develops dramatically. The umbilical cord blood makes contacts with all fetal tissues and is related to the physiological or pathological states of the fetus. Moreover, umbilical cord blood plays a critical role in transport of oxygen/carbon dioxide and other materials between mother and fetus through the placenta, thereby effecting how the intrauterine environment (maternal and placental factors) influences the fetus. Therefore, qualitative and quantitative changes of some umbilical cord serum proteins (i.e. α-fetoprotein, adiponectin and leptin) are widely used for clinical diagnosis and therapeutic monitoring of fetal or neonatal disorders [1].

Proteomics, a high flux approach in new protein discovery, is playing an increasingly important role in the development of novel biomarkers for various diseases [2]. Previously, proteomics techniques have been extensively applied in the investigation of adult plasma/serum proteomes in normal and diseased states [3]. More recently, increasing interest in umbilical cord proteomics has emerged, in order to search for novel biomarkers of fetal and neonatal diseases [4].

The first challenge in proteomics studies is sample preparation. For plasma/serum based proteomics research, the depletion of high abundance proteins is of great importance in sample preparation. In human serum, protein concentrations are found in an extremely wide range, with several kinds of highly abundant proteins (i.e. albumin and IgG) making up more than 80% of the total protein contained in the serum [5], while a number of important biomarkers are found only in trace amounts. High abundance proteins influence proteomics studies in two ways: Firstly, in 2DE gel based proteomics studies, low abundance proteins are likely to be masked by high abundance proteins on the gel. Secondly, the sample amount used for the study tends to be limited, and since highly abundant proteins completely dominate the protein content, detection of low abundance proteins becomes extremely difficult. Therefore, several depletion kits have been developed to remove highly abundant proteins in human plasma/serum [6-9] for use in further proteomics research. However, most of the depletion approaches have only been tested and evaluated in adult serum or plasma, and little data about their usage and efficiency in fetal or umbilical cord serum/plasma depletion was available.

Depletion kits remove highly abundant proteins by binding to specific sites on the proteins. However, some proteins (i.e. albumin) in fetal, neonatal and umbilical cord serum are structurally different from those in adult serum [10] and the depletion efficiency of a method varies when the method is applied in different sample types [11]. Therefore, it comes to question whether the approaches which revealed high depletion efficiency in adult serum/plasma are adequate to remove highly abundant proteins in umbilical cord serum.

To the furthest of our knowledge, only two kits: ProteoPrep Top 20 protein immunoaffinity column (Sigma-Aldrich, Saint Louis, MO, USA) [12] and the Multiple affinity removal column system (Agilent, Santa Clara, CA, USA) [3], had been used for depletion of high abundance proteins from umbilical cord serum samples. These two kits were able to remove several highly abundant proteins from umbilical cord serum and were compatible with iTRAQ or LC-MS studies, respectively. However, the compatibility of these two kits with 2DE based proteomics studies and their depletion efficiencies remained untested.

The primary aim of the present study is to evaluate the efficiency of three commonly used commercial kits on depletion of high abundance proteins from umbilical cord serum, and to search for an effective and reproducible approach to umbilical cord serum sample preparation for further proteomics research.

## Results

### Evaluation of depletion efficiency and reproducibility of each protein depletion kit using umbilical cord serum

Several approaches have been developed to deplete high abundance proteins from adult plasma/serum. However, their usage and efficiency of depletion of highly abundant proteins from fetal or umbilical cord serum/plasma remains unknown. To set up a high abundance protein depletion method suitable for umbilical cord serum, we evaluated the three most commonly used depletion kits, testing for depletion efficiency, reproducibility, and the yield of depleted serum.

**Figure 1:**
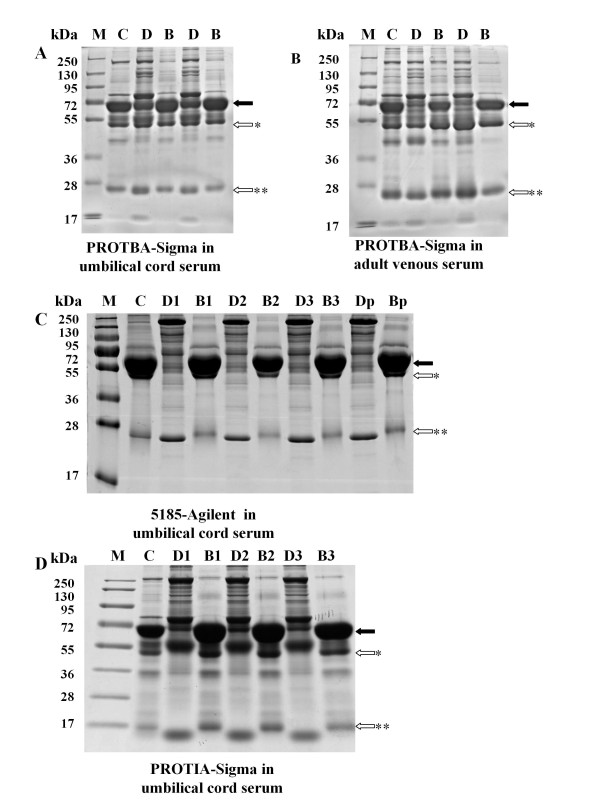
**Comparison of depletion efficiency and repeatability of different kits on umbilical cord serum proteins samples by 1D-SDS-PAGE**. Ten micrograms of crude, depleted or bound protein were loaded onto each lane and separated by 4-12% polyacrylamide gels. Proteins were visualized by Coomassie Blue staining. For examination of repeatability in umbilical cord serum depletion, the immunoaffinity columns (5185-Agilent and PROTIA-Sigma) were run three consecutive times with re-equilibration between depletion processes. However, for Blue Albumin and IgG Depletion Kit (PROTBA, Sigma-Aldrich, Saint Louis, MO, USA), serum samples were tested in two independent columns since its depletion efficiency was low even with a new column. A) Blue Albumin and IgG Depletion Kit (PROTBA, Sigma-Aldrich, Saint Louis, MO, USA) in umbilical cord serum depletion. B) Blue Albumin and IgG Depletion Kit (PROTBA, Sigma-Aldrich, Saint Louis, MO, USA) in adult venous serum depletion. C) Multiple Affinity Removal System (5185, Agilent, Santa Clara, CA, USA) in umbilical cord serum depletion. D) Immunoaffinity Albumin and IgG Depletion Kit (PROTIA, Sigma-Aldrich, Saint Louis, MO, USA) in umbilical cord serum depletion. Lanes: M: Prestained protein marker (Fermentas; 250, 130; 95, 72, 55, 36, 28, 17); C: Crude serum; D: Depleted serum; B: Bound proteins; D1-3: Depleted umbilical cord serum proteins of the first, second and third depletion process. B1-3: Bound proteins eluted from the column after the first, second and third depletion process. Dp: Depleted Proteins pooled by the three depletion processes. Bp: Bound proteins pooled by the three depletion processes Closed arrows: Protein band indicating albumin; Open arrows: Protein band indicating IgG; *heavy chain, and **light chain.

Immobilized dye based and immunoaffinity based methods are two major approaches in depletion of high abundance proteins. The Blue Albumin and IgG Depletion kit (PROTBA, Sigma-Aldrich, Saint Louis, MO, USA) is an immobilized dye based depletion kit which is highly efficient in depleting albumin and IgG from adult plasma/serum at a low cost. Therefore, we used the Blue Albumin and IgG Depletion kit (PROTBA, Sigma-Aldrich, Saint Louis, MO, USA) to measure the efficiency of albumin and IgG depletion from umbilical cord serum, using adult venous serum as control. In order to obtain the highest depletion efficiency, the lowest recommended amount of serum (25 μl) was loaded on the column. As shown in Figure 1A, after depletion by the PROTBA-Sigma kit, some of the highly abundant albumin and IgG bound to the column (Figure 1A, Lane B), however, a considerable amount still remained in the umbilical cord serum (Figure 1A, Lane D). On the contrary, the PROTBA-Sigma kit removed most of the albumin and IgG from adult venous serum (Figure 1B). This result suggests that the Blue Albumin and IgG Depletion kit could not deplete albumin and IgG effectively from umbilical cord serum. Since this immobilized dye based depletion kit did not show high efficiency, we then tested two immunoaffinity depletion kits using umbilical cord serum.

It has been reported that the Multiple Affinity Removal System (5185, Agilent, Santa Clara, CA, USA) was able to remove six kinds of highly abundant proteins (including albumin, IgG, a1-antitrypsin, IgA, transferrin, and haptoglobin) from human serum and could be used 200 times, repeatedly. To investigate the depletion efficiency and reproducibility of the 5185-Agilent system, we ran the system three times consecutively using an HPLC system. The 5185-Agilent system was equilibrated according to the manual guidelines prior to each depletion process. Next, samples were concentrated by ultrafiltration and the depleted or bound proteins were analyzed by SDS-PAGE either separately or as a pooled sample. As shown in Figure 1C, albumin and IgG were almost completely removed by the 5185-Agilent column and this high efficiency was maintained when the column was run a second and third time. The column not only removed albumin and IgG, but also cleared four other high abundance proteins, namely a1-antitrypsin, IgA, transferrin, and haptoglobin from the serum. This result demonstrated the high efficiency and good reproducibility of the 5185-Agilent column during umbilical cord serum depletion.

The second immunoaffinity kit we tested was the Immunoaffinity Albumin and IgG Depletion kit (PROTIA, Sigma-Aldrich, Saint Louis, MO, USA). It has been reported that this kit was able to remove 95% of albumin and IgG in human serum, but no data about its reproducibility was available. To evaluate the depletion efficiency and reproducibility of the PROTIA-Sigma kit, we used the kit three times consecutively, with re-equilibration between each depletion process. As shown in Figure 1D, the PROTIA-Sigma Kit effectively removed most of the albumin and IgG from the umbilical cord serum (Figure 1D, Lane D1, D2 and D3) in the first, second and third depletion process, and the depletion efficiency of each process was similar (Figure 1D, Lane B1, B2 and B3). This result suggested that the PROTIA-Sigma Kit was not only effective to remove high abundance proteins from umbilical cord serum, but could also be used repeatedly.

### Evaluation of yield for each protein depletion kit tested using umbilical cord serum

Besides depletion efficiency and repeatability, the protein yield post-depletion was another important factor regarding the compatibility of the depletion method with downstream analysis. Therefore, we measured the protein concentration of the depleted serum by Bradford Protein Assay (Bio-Rad, Hercules, CA, USA) to determine the protein yield after depletion by each of the kits.

Among the three kits, the PROTIA-Sigma kit had the highest load volume1 and produced the most depleted serum (Table 1). The PROTBA-Sigma Kit had the highest initial yield percentage and concentration of the three kits, likely because albumin and IgG were not completely removed by this kit. Since the 5185-Agilent system removed six kinds of highly abundant proteins from the serum, it resulted in the lowest yield and yield percentage. The primary protein concentrations of depleted sera by the PROTIA-Sigma and PROTBA-Sigma kits were both higher than 1 μg/μl, which is suitable for direct use in isoelectric focusing. The protein concentration of depleted serum by the 5185-Agilent kit was 2.871 μg/μl after concentration by a 5000 Da molecular mass cut-off centrifugal concentrator.

**Table 1:** Approximate Yield from Each Depletion Method

Method	Load Volume (μl)	Load (μg)	Yield (μg)	Yield %	Yield Concentration (μg/μl)
Blue Albumin and IgG Depletion Kit (PROTBA, Sigma-Aldrich)	25	1500	216	14.39	1.727
Multiple Affinity Removal System (5185, Agilent)	20	1200	133	11.08	2.871*
Immunoaffinity Albumin and IgG Depletion Kit (PROTIA, Sigma-Aldrich)	50	3000	340	11.34	1.513

*The protein concentration in the depleted solution of Multiple Affinity Removal System (Agilent) was detected after concentration by ultrafiltration using a 5000 Da molecular mass cut-off centrifugal concentrator.

In summary, the immunoaffinity based methods exceeded the immobilized dye based method in depletion efficiency, and both immunoaffinity based methods have high reproducibility. Since the yield for the depleted serum by the PROTIA-Sigma immunoaffinity kit was high with no additional concentrating or ion extracting required, we chose this kit for analysis by 2D electrophoresis to further evaluate its compatibility with downstream proteomics studies and to investigate whether high abundance protein depletion improves serum protein profiling patterns in 2DE gels.

### 2DE of crude and depleted umbilical cord serum samples

Depletion of high abundance proteins is expected to improve the serum protein profiling pattern of 2DE gels by enabling visualization of protein spots that were previously masked by the highly abundant proteins and by increasing the low abundance protein load. However, whether the depleted serum can be directly used for 2DE based proteomics studies remains unknown since depleted serum contains additional ions which may influence isoelectric focusing (IEF). Therefore, to determine the compatibility of the depletion method with 2DE based proteomics, depleted umbilical cord serum samples from the PROTIA-Sigma kit were selected for analysis by 2D gel electrophoresis, using crude serum samples as comparison.

**Figure 2:**
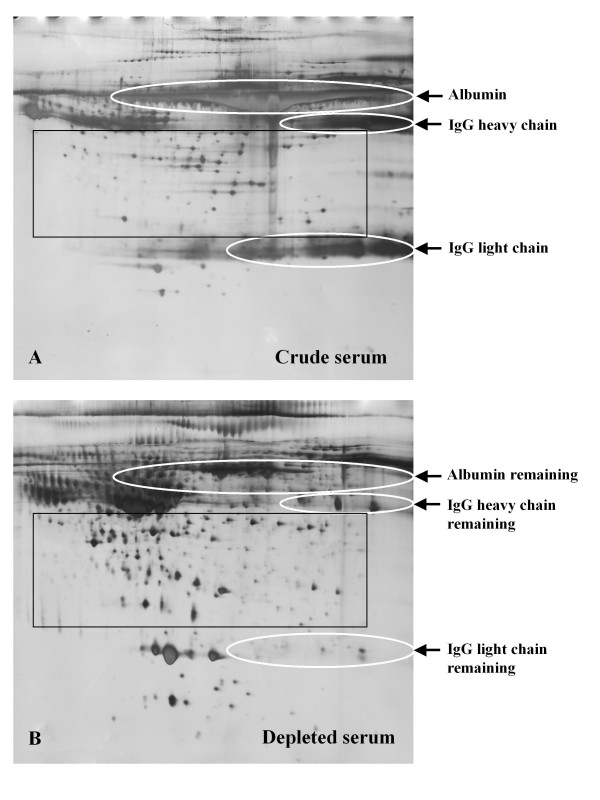
**Two Dimensional Electrophoresis Gel image of crude and depleted umbilical cord serum samples**. For each gel, 150 μg of protein was separated over pH range 4-7 (24 cm strips) and 12.5% SDS-polyacrylamide gel. The gel was visualized by silver staining. The Immunoaffinity Albumin and IgG Depletion Kit (PROTIA, Sigma-Aldrich, Saint Louis, MO, USA) Kit was used to deplete albumin and IgG in the umbilical cord serum. Removal of these proteins improved the resolution in the albumin and IgG areas on the gel and increased the intensity of low abundance proteins. A) Two Dimension Electrophoresis Gel image of crude umbilical cord serum. B) Two Dimension Electrophoresis Gel image of depleted umbilical cord serum by Immunoaffinity Albumin and IgG Depletion Kit (PROTIA, Sigma-Aldrich, Saint Louis, MO, USA). Circles: regions of gel containing albumin, heavy and light chain of IgG before (panel A) and after depletion (panel B). Rectangle: used to highlight different protein patterns in the two groups, indicating a large number of protein spots that emerged in the depleted gel.

As shown in Figure 2, albumin and IgG emerged in a large smeared protein pattern, masking proteins in a substantial area (Figure 2A, circles) on crude serum 2DE gels. Depletion of albumin and IgG by the PROTIA-Sigma kit improved the quality of 2DE gels in two ways: firstly, depletion of albumin and IgG resulted in improved separation within these regions of the gel and the emergence of several new spots (Figure 2B, circles). Additionally, the depletion of the high abundance proteins increased the load of low abundance proteins. Albumin and IgG represent about 80% of the total serum protein content and the PROTIA-Sigma kit was able to remove more than 90% of this albumin and IgG from the umbilical cord serum. Therefore, the loading capacity of low abundance proteins increased after protein depletion and many new protein spots emerged on the depleted serum 2DE gels (Figure 2B, Rectangle).

Image analysis using ImageMaster was performed on triplicate samples of crude and depleted serum 2DE gels. Since the protein patterns between crude and depleted serum gels were variable, matching was performed within groups. For the crude group, 411 ± 18 spots were detected while for the depleted group, 757 ± 43 spots were detected. The matching rate between gels in the crude serum group was 62.2 ± 4.3% and that in the crude group was 75.12 ± 3.9%.

**Figure 3:**
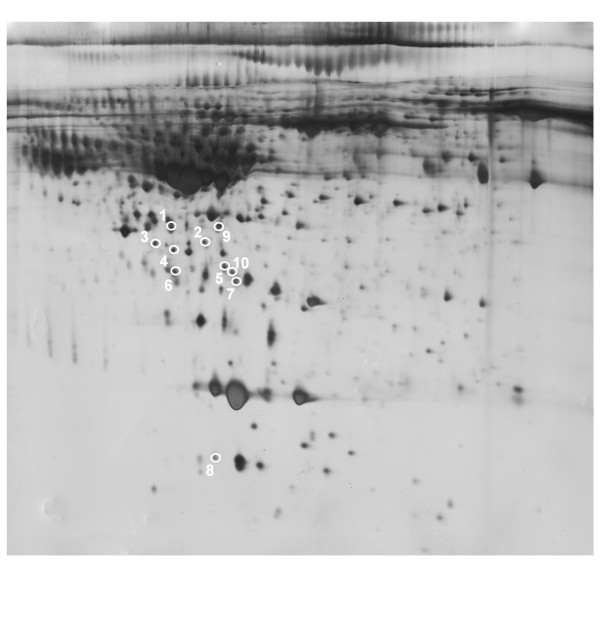
**Identification of protein spots unique to depleted umbilical cord serum gels by the Immunoaffinity Albumin and IgG Depletion kit (PROTIA, Sigma-Aldrich, Saint Louis, MO, USA)**. Circles: Ten spots unique to the depleted gels were selected for MALDI-TOF/TOF MS identification. Gel image is the same as in Fig 2B. The numbers correspond to the protein numbers in Table 2.

Next, ten unique spots from the depleted gels were randomly chosen for identification by MALDI-TOF/TOF MS, with selected protein spots indicated in Figure 3 (Gel image in Figure 3 is the same as in Figure 2B). Eight of ten spots were successfully identified by MS, with identifications determined according to the highest Mascot MS/MS score (Table 2). The majority of the spots, including Zinc-alpha-2-glycoprotein, Hematopoietic lineage cell-specific protein, Tubulin alpha chain-like 3, Apolipoprotein E, Tropomyosin beta chain, Sorcin, Tetranectin, were low abundance proteins. These protein spots could not have been directly detected in the crude umbilical cord serum 2DE gels due to interference by high abundance proteins, but became apparent in the depleted serum 2DE gels.

**Table 2:** MALDI-TOF/TOF MS identification of selected protein spots which were unique to the depletion gels.

No	Protein ID	Protein name	Mw (kDa)	pI	Mascot MS/MS score	Sequence coverage (%)
1	P25311 ZA2G_HUMAN	Zinc-alpha-2-glycoprotein	34.2	5.6	93	4
2	P14317 HCLS1_HUMAN	Hematopoietic lineage cell specific protein	53.9	4.7	229	12
3	P01024 CO3_HUMAN	Complement C3	187.1	6.0	53	1
4	A6NHL2 TBAL3_HUMAN	Tubulin alpha chain-like 3	49.9	5.7	64	16
5	P02649 APOE_HUMAN	Apolipoprotein E	36.2	5.5	88	35
6	P07951 TPM2_HUMAN	Tropomyosin beta chain	32.9	4.5	69	8
7	P30626 SORCN_HUMAN	Sorcin	21.7	5.3	185	21
8	P05452 TETN_HUMAN	Tetranectin	22.6	5.8	57	10

Spots with the numbers 9 and 10 could not be successfully identified.

## Discussion

In the present study, we have compared three commercially available depletion kits on umbilical cord serum depletion, regarding depletion efficiency, reproducibility, protein yield and compatibility with downstream proteomics research. Results have demonstrated that the immunoaffinity depletion kits exceeded the immobilized dye based kit in depletion efficiency when used on umbilical cord serum. The depleted umbilical cord serum produced by the Immunoaffinity Albumin and IgG Depletion kit (PROTIA, Sigma-Aldrich, Saint Louis, MO, USA) could be directly applied in isoelectric focusing (IEF) and improved 2DE gel quality for low abundance protein detection.

The first important aspect in evaluating a depletion kit is depletion efficiency. The PROTBA-Sigma Kit mainly contains Cibacron Blue and Protein A/G. Cibacron Blue has been reported to bind human serum albumin by interacting with the bilirubin-binding site(s) and the fatty acid-anion-binding sites [13]. Protein A has been reported to bind the Fc portion of IgG, while Protein G binds to both the Fc and Fab portion of IgG [14]. As a dye-based depletion kit, the PROTBA-Sigma kit demonstrated considerable depletion efficiency in adult plasma/serum in previous studies, but albumin in umbilical cord serum could not be completely removed by this kit in the present study. The low efficiency of this kit in umbilical cord serum depletion may be due to structural difference between fetal and adult albumins. In a previous study, Wallace S [10] identified that the structure of neonatal albumin (similar to fetal albumin) was different from adult albumin due to variable amino acid composition. In another study, Ingrid Miller *et al *[11] showed the affinity of Cibacron Blue F3GA, an immobilized dye matrix, was variable for albumin of different species due to the structural variations of the proteins. Therefore, based on the data from these two previous studies and the present study, we infer that structural alterations caused by amino acid variations of fetal albumin leads to the low interaction strength between fetal albumin and blue matrix in the PROTBA-Sigma kit.

Conversely, both immunoaffinity based kits, the 5185-Agilent kit and the PROTIA-Sigma kit, had higher efficiency in umbilical cord serum depletion. Although structurally different, fetal albumin is immunologically similar to adult albumin [15]. Therefore, as expected, the immunoaffinity based depletion kits had comparable efficiency when applied to adult and umbilical cord serum depletion.

The second aspect when analyzing a depletion method is reproducibility. The protein amount used in proteomics studies is large with replicate experiments often required. Since the protein yield produced by a single depletion process is typically not enough for further analysis, the reproducibility of a kit is very important for preparation of a large scale sample. In a recent study on umbilical cord serum proteomics [3], the Multiple Affinity Removal System (Agilent, Santa Clara, CA, USA) was used to deplete high abundance proteins from umbilical cord serum. The one-dimensional SDS-PAGE analysis in this article demonstrated that the depletion efficiency was considerable, but reproducibility of the kit was not reported. Therefore, we extended this evaluation of the 5185-Agilent kit as well as the PROTIA-Sigma kit to determine reproducibility, in order to maximize the yield for further proteomics studies. Results from this study indicate that both immunoaffinity based kits had high reproducibility, with similar depletion efficiencies occurring after the columns were used for three consecutive repeat experiments.

The third factor in assessing a depletion kit is protein yield. Since the sample volume and protein amount for IEF should both be within fixed ranges, appropriate protein concentrations of depleted serum is required for 2DE based proteomics. According to previous studies, the protein concentration of the loading sample for silver stained 2DE gels should not be less than 0.5 μg/μl, and the concentration of the loading sample for Coomassie Blue stained 2DE gels should be even higher. Therefore, the depleted sera by the PROTBA-Sigma and PROTIA-Sigma kits, as well as the concentrated sera produced by the 5185-Agilent kit were all within the appropriate protein concentration range. Both the serum load and the protein yield were highest for the PROTIA-Sigma kit, indicating that it was most suitable for large scale protein preparation. It should be noted that although the protein yield percentage of the PROTBA-Sigma Kit was in fact highest, this was likely due to contamination by albumin and IgG that was not depleted.

The fourth and most important factor in evaluating a depletion kit is whether the resulting serum can be used in downstream proteomics studies and if it can improve the quality of these studies. The PROTBA-Sigma kit was excluded due to its low depletion efficiency and so the choice remained between the two immunoaffinity kits. Of the two kits, the PROTIA-Sigma kit was chosen for two reasons: firstly, the usage of the 5185-Agilent kit in umbilical cord serum had been previously reported and so if the PROTIA-Sigma kit proved successful, it would provide a new choice for researchers. Secondly, the depleted serum from the PROTIA-Sigma kit could be used for isoelectric focusing (IEF) directly since the wash solution contained fewer ions. Depletion improved 2DE gel quality by two aspects. Firstly, since the majority of albumin and IgG were depleted, protein spots which were masked in the crude gels became detectable. Secondly, the intensity of many low abundance proteins increased because they now represented a larger proportion of the total sample amount.

To date, many immunoaffinity based depletion kits have been designed for removal of two, six, twelve, or twenty types of highly abundant proteins from serum. Therefore, another question when choosing a depletion kit for serum/plasma proteomics is how many kinds of high abundance proteins should be depleted [16]. The choice of depletion method depends on the design of the study, the experimental method, and the balance between the removal of highly abundant proteins and the loss of associated low abundance proteins. Albumin and IgG represent about 80% of the total protein content in umbilical serum, so removal of albumin and IgG is expected to increase the low abundance protein load by approximately 4-5 times. In the present study, we identified eight protein spots unique to depleted serum gels, most of them low abundance proteins. Therefore, removal of the two most abundant proteins (albumin and IgG) is adequate for detection of primarily low abundance biomarkers. However, removal of more kinds of highly abundant proteins may be needed for further enrichment of some proteins naturally found in trace amounts. In that case, the 5185-Agilent system may play an important role, since it can remove six kinds of high abundance proteins with high specificity [17].

## Conclusions

Our study compared three high abundance protein depletion kits on umbilical cord serum sample preparation for proteomics, regarding their depletion efficiency, reproducibility, yield and compatibility with downstream proteomics studies. The immunoaffinity based depletion methods exceeded the immobilized dye based method in depletion efficiency. Both of the immunoaffinity based kits, PROTIA-Sigma and 5185-Agilent revealed high reproducibility and appropriate protein yield for further proteomics analysis (although an additional protein concentrating step was required for the 5185-Agilent kit). Based on 2DE and MS results, the PROTIA-Sigma Kit dramatically improved 2DE gel quality by increasing low abundance protein intensity, and thus serves as a useful tool in novel biomarker discovery.

## Methods

### Sample collection

Umbilical cord venous blood samples were provided by two healthy pregnant women after placenta delivery. Venous blood samples from two healthy adults were chosen as control for comparison of depletion efficiency between adult serum and umbilical cord serum. The samples were collected in sterile vacuum blood collection tubes and placed at room temperature (25°C) for one hour to allow the blood to clot. After blood coagulation, sera were separated by centrifugation at 720 g for 15 min at 4°C, frozen in aliquots of 100 μl, and stored at -80°C until analysis. The study was approved by the research ethical committee of The First Affiliated Hospital of Sun Yat-sen University and informed consent was provided by all volunteers.

### Depletion of high abundance proteins from umbilical cord serum by three kits

Three depletion kits were used in the present study: the ProteoPrep Blue Albumin and IgG Depletion Kit (PROTBA, Sigma-Aldrich, Saint Louis, MO, USA), the Agilent Multiple Affinity Removal System (5185, Agilent, Santa Clara, CA, USA) and the ProteoPrep Immunoaffinity Albumin and IgG Depletion Kit (PROTIA, Sigma-Aldrich, Saint Louis, USA).

The depletion processes were performed in accordance with manuals as follows:

(1) For the PROTBA-Sigma Kit, 25 μl of serum was loaded onto the equilibrated column and incubated two consecutive times and the "twice depleted" serum was collected. Then, the bound proteins (albumin and IgG) were eluted by the Protein Extraction Reagent Type 4.

(2) For the 5185-Agilent column, 20 μl of serum was diluted in 80 μl Buffer A prior to completion of the depletion process. After column equilibration, 100 μl of diluted serum was loaded onto the column at a flow rate of 250 μl/min. Buffer A was then injected at a flow rate of 250 μl/min and fractions between 1.5-4.5 min were collected. Next, bound proteins were eluted with Buffer B at a flow rate of 1000 μl/min. Buffer A was used to regenerate the column for further use.

(3) For the PROTIA Kit, 50 μl of serum was diluted in 50 μl of Equilibration Buffer before performing the depletion process. After column equilibration, 100 μl of diluted serum was loaded onto the column and incubated two consecutive times. The "twice depleted" serum was then collected and the bound proteins were eluted by Protein Extraction Reagent Type 4.

### Protein assays

Total protein concentration of crude serum, depleted samples, and eluted proteins were determined by Bradford protein assay according to the manufacturer's instructions (Bio-Rad, Hercules, CA, USA), using bovine serum albumin (BSA) as a standard. The depleted and eluted proteins from 5185-Agilent kit were concentrated using 5000 Da molecular mass cut-off centrifugal concentrators (Millipore, Billerica, MA, USA) prior to determination of protein concentration. Samples were then stored at -80°C until further analysis.

### 1D-SDS-PAGE

The depletion efficiency of each approach was evaluated by SDS-PAGE. Ten micrograms of crude, depleted, and bound proteins from each approach were loaded into each lane of 12% resolving and 4% stacking polyacrylamide gels (GE Healthcare, Waukesha, WI, USA) and electrophoresed through a Bio-Rad system (Bio-Rad, Hercules, CA, USA) using the Laemmli SDS buffering system (25 mM Tris-base, 192 mM glycine, 0.1%SDS). The gels were stained with Coomassie Blue (GE Healthcare, Waukesha, WI, USA) overnight according to the manufacturer's instructions.

### Two-dimensional Gel Electrophoresis

For 2DE analysis, 150 μg of depleted or crude umbilical cord serum protein samples were diluted in sample buffer consisting of 50 mM Tris-HCl (pH 8.5), 7 M urea, 2 M thiourea, 2% CHAPS, 0.3% dithiothreitol (DTT), 0.5% IPG buffer (pH 4-7 linear), and 10 μl of a protease inhibitor mixture (Sigma-Aldrich, Saint Louis, MO, USA) to a final volume of 450 μl. Protein samples were applied on immobilized pH 4-7 linear gradient IPG strips (24 cm) (Bio-Rad, CA, USA) and active rehydration was performed at 30 V for 6 h and 60 V for 6 h. Focusing was performed using an Ettan IPGphor III IEF system (GE Healthcare, Waukesha, WI, USA) at 200 V for 2 h, 500 V for 2 h, 1000 V for 2 h, 5000 V for 2 h, after which the voltage was gradually increased to 10000 V for 85000 Vhs and kept at 500 V for 5 h.

After focusing, strips were equilibrated for 15 min in 75 mM Tris-HCl (pH 8.8), 6 M urea, 2% w/v SDS, 29.3% v/v glycerol, and 1.0% DTT, followed by a 15 min incubation in the same buffer containing 2.5% iodoacetamide in place of DTT. Second-dimension electrophoresis was performed on a 12.5% polyacrylamide gel, using an Ettant DALT Six system (GE Healthcare, Waukesha, WI, USA). Each gel was run at 2 w for 1 h at 15°C, and then increased to 15 w until the tracking dye migrated to within 1 cm of the bottom of the gel.

### Protein Visualization and Computer Analysis of Protein spots

2D gels were fixed in 50% methanol (containing 5% acetic acid) for 2 h and visualized by silver staining using a modified protocol of Grit Nebrich et al [18]. Briefly, after washing three times with distilled water, the gels were sensitized with sodium thiosulphate (0.02%w/v) for 2 min, and then washed twice (one minute each) with distilled water. Next, the gels were incubated in 0.1% silver nitrate solution for 30 minutes. After 3 rinses, the gels were developed with 2% sodium carbonate solution (containing 0.05% formalin) for 2-10 min and terminated with 5% acetic acid. After incubation in 5% acetic acid for 20 min, the gels were transferred into distilled water and scanned using the ImageScanner system (GE Healthcare, Waukesha, WI, USA) combined with LabScan software (GE Healthcare, Waukesha, WI, USA). All gel images were analyzed using ImageMaster 2D Platinum software (GE Healthcare, Waukesha, WI, USA). Three 2D gels from the depleted sample and three from the crude serum were analyzed. Gel images from each group were edited, and spots were matched. A unique identification number was assigned to matching spots on different gels. Normalization of the spot intensities was conducted according to the total optical density of the gel.

### In-gel tryptic digestion

Spots from 2DE gels selected for further analysis were cut using a blade and gel pieces were transferred to microfuge tubes. After rinsing with distilled water and destaining with potassium ferricyanide and sodium thiosulfate, the gel pieces were dehydrated in 100% acetonitrile. 2 μL (25 ng/μl) of modified porcine trypsin in 25 mM ammonium bicarbonate, pH 8, was added to each sample and incubated at 37°C overnight. The trypsin solutions were collected and the remaining peptides were extracted from the gel pieces by incubation in 0.1% Trifluoroacetic acid/60% acetonitrile for 15 min prior to drying in a vacuum centrifuge.

### MALDI-MS and MS/MS analysis

MALDI-TOF/TOF MS measurements were performed on a Bruker Ultraflex III MALDI-TOF/TOF MS (Bruker Daltonics, Leipzig, Germany) operating in reflectron mode with 20 kV accelerating voltage and 23 kV reflecting voltage. A saturated solution of α-cyano-4-hydroxycinnamic acid in 50% acetonitrile and 0.1% trifluoroacetic acid was used as the matrix. One microliter of the matrix solution and sample solution at a ratio of 1:1 was applied onto the Score384 target well. By routine, a standard peptide calibration mix in the mass range 800-3200 Da (Bruker Daltonics, Leipzig, Germany) was analyzed for external calibration of the mass spectrometer. The calibration mix contained: Angiotensin II, Angiotensin I, Substance P, Bombesin, ACTH clip 1-17, ACTH clip 18-39, and Somatostatin 28. A series of eight samples were spotted around one external calibration mixture. The SNAP algorithm (S/N threshold: 5; Quality Factor Threshold: 30) in FlexAnalysis 3.0 was used to pick up the 100 most prominent peaks in the mass range m/z 700-4000. The subsequent MS/MS analysis was performed in a data-dependent manner, and the 5 most abundant ions fulfilling certain preset criteria (S/N higher than 3 and Quality Factor higher than 30) were subjected to high energy CID analysis. The collision energy was set to 1 keV, and nitrogen was used as the collision gas.

### Database searching

Peptide mass fingerprints (PMFs) were searched using the program Mascot 2.1 (Matrix Science Ltd) against the SwissProt database (version 20091028, 510076 sequences). The search parameters were as follows: trypsin digestion with one missed cleavage; carbamidomethyl modification of cysteine as a fixed modification and oxidation of methionine as a variable modification; peptide tolerance maximum, ±0.3 Da; MS/MS tolerance maximum, ±50 ppm; peptide charge, +1; monoisotopic mass. p < 0.05 is for a local PMF search. For unambiguous identification of proteins, more than 5 peptides must be matched for a PMF search.

## Competing interests

The authors declare that they have no competing interests.

## Authors' contributions

BL collected clinical serum samples, performed the depletion experiments, carried out 2DE experiments, and drafted the manuscript. FHQ participated in the 2DE experiments. CV revised the manuscript. YX participated in the depletion experiments. MZZ carried out the TOF/TOF MS identification of differential protein spots. YXW participated in the collection of samples. JN and ZLW supervised the work and revised the manuscript. All authors have read and approved the manuscript.

## References

[B1] Kyriakakou M Malamitsi-Puchner A Militsi H Boutsikou T Margeli A Hassiakos D Kanaka-Gantenbein C Papassotiriou I Mastorakos G Leptin and adiponectin concentrations in intrauterine growth restricted and appropriate for gestational age fetuses, neonates, and their mothers Eur J Endocrinol 2008 158 343 348 10.1530/EJE-07-0692 18299467

[B2] Anderson NL Anderson NG The human plasma proteome: history, character, and diagnostic prospects Mol Cell Proteomics 2002 1 845 867 10.1074/mcp.R200007-MCP200 12488461

[B3] Song HJ Zhang P Guo XJ Liao LM Zhou ZM Sha JH Cui YG Ji H Liu JY The proteomic analysis of human neonatal umbilical cord serum by mass spectrometry Acta Pharmacol Sin 2009 30 1550 1558 10.1038/aps.2009.140 19890362 PMC4003003

[B4] Page NM Kemp CF Butlin DJ Lowry PJ Placental peptides as markers of gestational disease Reproduction 2002 123 487 495 10.1530/rep.0.1230487 11914111

[B5] Urbas L Brne P Gabor B Barut M Strlic M Petric TC Strancar A Depletion of high-abundance proteins from human plasma using a combination of an affinity and pseudo-affinity column J Chromatogr A 2009 1216 2689 2694 10.1016/j.chroma.2008.10.104 19010473

[B6] Tanaka Y Akiyama H Kuroda T Jung G Tanahashi K Sugaya H Utsumi J Kawasaki H Hirano H A novel approach and protocol for discovering extremely low-abundance proteins in serum Proteomics 2006 6 4845 4855 10.1002/pmic.200500774 16878292

[B7] Bellei E Bergamini S Monari E Fantoni LI Cuoghi A Ozben T Tomasi A High-abundance proteins depletion for serum proteomic analysis: concomitant removal of non-targeted proteins Amino Acids in press 10.1007/s00726-010-0628-x 20495836

[B8] Govorukhina NI Reijmers TH Nyangoma SO van der Zee AG Jansen RC Bischoff R Analysis of human serum by liquid chromatography-mass spectrometry: improved sample preparation and data analysis J Chromatogr A 2006 1120 142 150 10.1016/j.chroma.2006.02.088 16574134

[B9] Björhall K Miliotis T Davidsson P Comparison of different depletion strategies for improved resolution in proteomic analysis of human serum samples Proteomics 2005 5 307 317 10.1002/pmic.200400900 15619298

[B10] Wallace S Altered plasma albumin in the newborn infant Br J Clin Pharmacol 1977 4 82 85 402927 10.1111/j.1365-2125.1977.tb00673.x PMC1428993

[B11] Miller I Gemeiner M An electrophoretic study on interactions of albumins of different species with immobilized Cibacron Blue F3G A Electrophoresis 1998 19 2506 2514 10.1002/elps.1150191425 9820975

[B12] Colquhoun DR Goldman LR Cole RN Gucek M Mansharamani M Witter FR Apelberg BJ Halden RU Global screening of human cord blood proteomes for biomarkers of toxic exposure and effect Environ Health Perspect 2009 117 832 838 19478969 10.1289/ehp.11816 PMC2685849

[B13] Leatherbarrow RJ Dean PD Studies on the mechanism of binding of serum albumins to immobilized cibacron blue F3G A Biochem J 1980 189 1 27 34 7458904 10.1042/bj1890027 PMC1161914

[B14] Andrew SM Titus JA Purification of immunoglobulin G Curr Protoc Immunol 2001 Chapter 2 Unit 2.7 18432771 10.1002/0471142735.im0207s21

[B15] Gitzelmann-Cumarasamy N Gitzelmann R Wilson KJ Kuenzle CC Fetal and adult albumins are indistinguishable by immunological and physicochemical criteria Proc Natl Acad Sci USA 1979 76 2960 2963 10.1073/pnas.76.6.2960 111248 PMC383730

[B16] Roche S Tiers L Provansal M Seveno M Piva MT Jouin P Lehmann S Depletion of one, six, twelve or twenty major blood proteins before proteomic analysis: the more the better? J Proteomics 2009 72 945 951 10.1016/j.jprot.2009.03.008 19341827

[B17] Bellei E Bergamini S Monari E Fantoni LI Cuoghi A Ozben T Tomasi A High-abundance proteins depletion for serum proteomic analysis: concomitant removal of non-targeted proteins Amino Acids 2011 40 1 145 56 10.1007/s00726-010-0628-x 20495836

[B18] Nebrich G Herrmann M Sagi D Klose J Giavalisco P High MS-compatibility of silver nitrate-stained protein spots from 2-DE gels using ZipPlates and AnchorChips for successful protein identification Electrophoresis 2007 28 10 1607 14 10.1002/elps.200600656 17447244

